# Solving the Telomere Replication Problem

**DOI:** 10.3390/genes8020055

**Published:** 2017-01-31

**Authors:** Laetitia Maestroni, Samah Matmati, Stéphane Coulon

**Affiliations:** Aix Marseille Univ, CNRS, INSERM, Institut Paoli-Calmettes, CRCM, Equipe labélisée Ligue Contre le Cancer, 13273 Marseille, France; laetitia.maestroni@inserm.fr (L.M.); samah.matmati@inserm.fr (S.M.)

**Keywords:** telomere, telomere replication, G-quadruplex, T-loop, TERRA

## Abstract

Telomeres are complex nucleoprotein structures that protect the extremities of linear chromosomes. Telomere replication is a major challenge because many obstacles to the progression of the replication fork are concentrated at the ends of the chromosomes. This is known as the telomere replication problem. In this article, different and new aspects of telomere replication, that can threaten the integrity of telomeres, will be reviewed. In particular, we will focus on the functions of shelterin and the replisome for the preservation of telomere integrity.

## 1. Introduction

**Figure 1:**
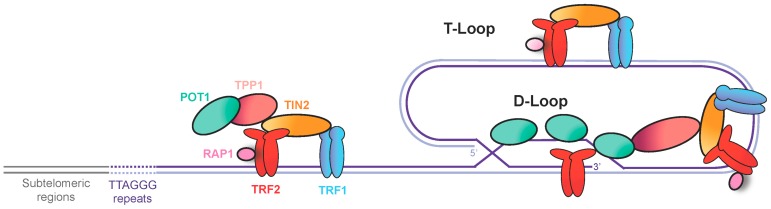
The vertebrate shelterin complex. Telomeric repeat binding factors 1 and 2 (TRF1 and TRF2) bind to double-stranded telomeric DNA and recruit other components of shelterin: TRF1-interacting nuclear protein 2 (TIN2), the human ortholog of the yeast repressor/activator protein 1 (RAP1), and tripeptidyl peptidase 1 (TPP1). Protection of telomeres 1 (POT1) protein binds to the telomeric single-stranded DNA. Shelterin promotes the fold-back of telomeric DNA into a T-loop structure through formation of a D-loop.

The linearization of chromosomes in eukaryotes allows the shuffling of alleles between homologous chromosomes during meiosis [1,2]. The extremities of linear chromosomes, named telomeres, are a consequence of this evolutionary change in eukaryotes, and could be considered as the “Achilles heel” of a chromosome. Mammalian telomeres contain several kilobases (5–15 kb) of tandemly-repeated sequences (5′-TTAGGG-3′) that terminate with 30–400 nucleotides of single-strand DNA on the G-rich strand, called the 3′ overhang. Mammalian telomeric DNA can fold back into a T-loop structure, in which the 3′ overhang invades the duplex DNA of repeated sequences. TTAGGG sequences are specifically recognized by several telomeric proteins, that together form the shelterin complex [3] (Figure 1). The telomeric repeat binding factor 1 and 2 (TRF1 and TRF2) bind to double-strand telomeric tracks, while the protection of telomeres 1 protein (POT1) binds to the 3′ overhang. TRF1 and TRF2 recruit the other components of shelterin: TRF2 and TRF1-interacting nuclear protein 2 (TIN2), the human ortholog of the yeast repressor/activator protein 1 (RAP1), and TPP1, also called TINT1, PTOP and PIP1. One function of shelterin is to protect the physical chromosome ends by inhibiting the DNA damage response pathways, including activation of the checkpoint kinases ataxia-telangiectasia mutated (ATM) and ATM- and Rad3-Related (ATR), as well as classical and alternative non-homologous end joining (NHEJ) [4]. Dysfunctional shelterin, which can be caused by the downregulation of TRF2, may eventually lead to telomere fusions, p53-dependent cell cycle arrest in G_1_, and senescence [3]. In the absence of p53 function, telomere fusions trigger cell death during mitosis [5]. Thus, shelterin is essential for the stability of the linear genomes.

At each cell division, the telomeres shorten because of the incomplete replication of the linear DNA molecules by the conventional DNA polymerases. This is called the end replication problem [6]. This is specifically due to the resection and fill-in reaction during the synthesis of the telomere leading-strand [7,8]. To circumvent this irremediable telomeric loss, shelterin also functions to recruit a reverse transcriptase called telomerase, that is able to elongate the 3′ overhang by the addition of telomeric repeats. The telomerase is a ribonucleoprotein minimally comprised of the catalytic subunit (TERT, telomerase reverse transcriptase) and its intrinsic RNA (TERC, telomerase RNA component). The TERT subunit is recruited to telomeres through its association with TPP1 during the S phase of the cell cycle [9,10], and is inhibited by the CTC1–STN1–TEN1 complex (CST) that acts as a terminator of telomerase activity [11]. In addition, the CST complex stimulates the DNA polymerase α/primase for the synthesis of the complementary strand [12]. In the absence of telomerase activity, telomeres undergo gradual shortening, leading to the inhibition of proliferation via either replicative senescence [13], or more rarely, by apoptosis (depending on the cellular context). One of the hallmarks of cancer cells is their ability to proliferate indefinitely. Indeed, cancer cells counteract the shortening of telomeres either by re-activating telomerase, or by using a homologous recombination telomere-copying mechanism, known as alternative lengthening of telomeres (ALT).

In mammalian cells, the majority of telomeres are replicated throughout the S phase [14,15]. In contrast, in budding and fission yeasts, telomeric DNA replication occurs at the end of the S phase [16,17]. Short telomeres, however, replicate earlier [18,19], and this is facilitated by Tel1 in budding yeast [20]. Remarkably, when the global replication program is perturbed, telomeres are also replicated earlier, modifying the telomere length equilibrium [21,22]. This underscores the importance of the accurate timing of telomeric sequence replication, which is a prerequisite for telomere homeostasis and telomerase control in yeast. Moreover, it becomes clear that any obstacles that impede replication fork progression will generate a stress which alters telomere length homeostasis. This defines the telomere replication problem [23]. Out of the entire genome, telomeres are one of the most difficult regions to replicate because they encompass multiple difficulties for replication. In this essay, we will review different and new aspects of telomere replication that threaten the integrity of telomeres, examining how the replication machinery and shelterin deal with this challenge.

## 2. Telomeres, a Source of Replication Stress

Telomere replication mostly proceeds unidirectionally, from a centromere-proximal origin. DNA two-dimensional gel electrophoresis analysis (2D-gel) in yeast has demonstrated that replication forks naturally slow and eventually stall, as they approach telomeric chromatin [23,24,25]. If stalling persists, the fork may collapse and the newly generated double-strand break triggers a restart mechanism through homologous recombination, risking the loss of genetic material. In mammals, replication stress leads to telomere aberrations characterized by abnormal structures on metaphase chromosomes that can be visualized by fluorescence in situ hybridization (FISH) [26]. The nature of these aberrant structures has not been clearly established, but they are thought to arise from partially replicated and broken telomeres, or may even represent entangled telomeres. Therefore, telomeres are fragile sites that are hard to replicate and can generate a stress when replicated.

What causes replication fork stalling at telomeres? Heterochromatin, G-rich regions that are prone to forming secondary structures such as G-quadruplex (G4), T-loop super-structures, telomere repeat containing RNA (TERRA) transcription including R-loop and RNA:DNA hybrids, as well as telomere compaction and telomere attachment to the nuclear envelope, are diverse sources of endogenous blocks that impede the progression of the fork through telomeric tracts. Although telomere-bound proteins have been thought to impede replication fork progression [27], it has been subsequently established that TRF1, TRF2, and their fission yeast counterpart Taz1 are required for promoting the efficient replication of telomeres and preventing fork stalling [23,26,28,29]. This highlights the active role played by shelterin in ensuring efficient replication. Although many of the molecular mechanisms involved in this process remain to be elucidated, we will describe the known interactions between TRF1 and TRF2, and several partners (helicase or nuclease), that promote the efficient replication of telomeres. Interestingly, TRF1 and TRF2 also associate with the Timeless protein, a component of the fork protection complex (FPC) [30]. The FPC is a part of the replisome, and ensures proper replication fork pausing and the smooth passage of the replication forks at hard-to-replicate regions, including telomeres (reviewed in Leman et al., 2012 [31]). Remarkably, timeless downregulation or deletion of the timeless ortholog *swi1* lead to shortened telomeres in human cells and in fission yeast, respectively [30,32]. In fission yeast, Swi1 ensures the correct replication of telomeres, presumably by maintaining the fork in a natural conformation to prevent fork collapse and the instability of telomeric repeats [33]. These observations show, first, that replisome integrity is important for telomere maintenance, and second, that the replisome and shelterin act together to guarantee telomeric tract stability by preventing the stalling and collapse of replication forks. Here, we will present the numerous strategies that cells employ to overcome the telomere replication problem. How replisome and shelterin collaborate to ensure the proper replication of telomeres may represent a major area of investigation for the next decade.

## 3. Secondary Structures at Telomeres

### 3.1. G-Quadruplex Dissolution by Helicases

Four guanines can associate through Hoogsteen hydrogen bonding to form a planar G-quartet, stabilized by a monovalent cation. Three or more G-quartets can stack on top of each other to form a G-quadruplex structure, referred to as G4. G-rich sequences, such as telomeric DNA, that contain four runs of at least three guanine bases, can form stable intramolecular G4. G4 formation is promoted in single-stranded DNA during DNA replication and transcription. Thus, G4 structures can constitute prominent barriers to replication fork progression, and are intrinsically recombinogenic and mutagenic, leading to the idea that G4 may promote chromosome instability [34]. At telomeres, G4 can form on the G-rich lagging strand template and cause fork arrest. If not properly resolved, G4 can eventually cause fork breakage and telomere loss. On the other hand, G4 might also participate in telomere protection when formed in the G-rich telomeric overhang [7,35].

**Figure 2:**
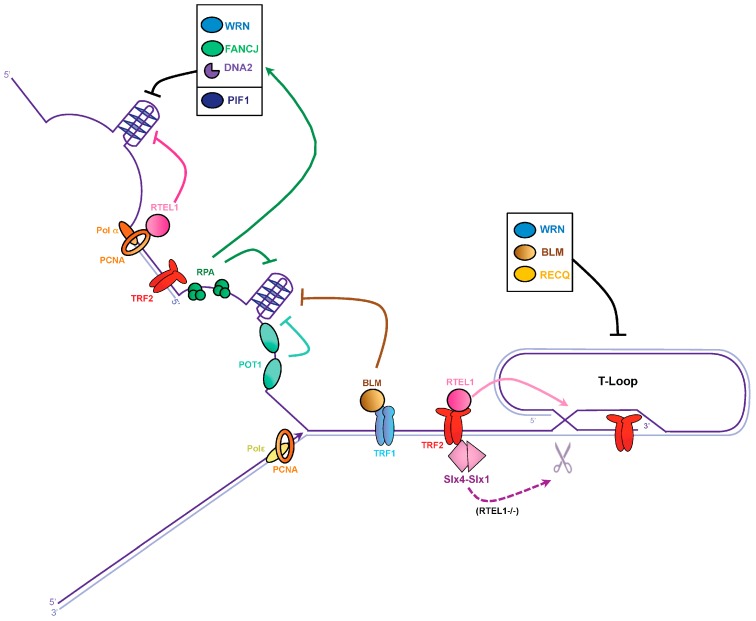
Dealing with G-quadruplexes and T-loops to avoid replication fork stalling at telomeres. Lagging and leading telomeres are replicated by DNA polymerase α and ε (Polα and Polε), respectively. TRF1 recruits the BLM helicase and proliferating cell nuclear antigen (PCNA) associates with the regulator of telomere elongation 1 (RTEL1) helicase to unwind G4 on the lagging strand. The single-strand DNA binding proteins replication protein A complex (RPA) and POT1 could prevent G4 formation at telomeres. Additionally, WRN, Fanconi anemia group J (FANCJ), and DNA2 may also contribute to G4 resolution, possibly through RPA stimulation, while PIF1 helicase might act on its own. T-loop disassembly is performed by RTEL1. In the absence of RTEL1, the SLX1–SLX4 nuclease might resolve T-loops. RECQ helicase members might also participate in T-loop resolution.

To avoid genetic instability and telomeric alterations, G4 must be unwound (Figure 2). Many helicases have been implicated in this process. These include the 3′–5′-directed RecQ helicase family, Werner syndrome RecQ like helicase (WRN), and Bloom syndrome RecQ like helicase (BLM) [36,37,38]. WRN and BLM defects are responsible for the high predisposition to cancer in people with Werner’s and Bloom’s syndromes, respectively. Cells lacking WRN exhibit a loss of the telomeric lagging strand, which contains the G-rich sequence capable of forming G4 structures [39,40]. WRN likely plays a major role in unwinding G4 during telomere replication, however, the molecular bases of WRN involvement in telomere replication are unknown. Recruitment of WRN at G4 sites could be mediated through interactions with several partners as RPA (replication protein A complex), PCNA (proliferating cell nuclear antigen), and Pol δ [41], as well as TRF2 [42]. Unlike WRN, a BLM-deficient cell line shows a fragile telomere phenotype, and this effect is epistatic with TRF1 depletion, suggesting that BLM facilitates telomere replication in a TRF1-dependent manner, presumably by removing G4 [26]. Indeed, BLM contains the FxLxP TRF1 binding motif and binds TRF1 in vitro [43]. Along the same lines, it has been more recently proposed that TRF1 recruits BLM in vivo to remove secondary structures such as G4, preventing lagging-telomere fragility [44]. Thus, the shelterin component TRF1 seems to play an active role in removing G4 formed on the lagging strand during replication of telomeric repeats, by recruiting the specific G4 helicase BLM.

Similarly to WRN and BLM helicases, RTEL1 (regulator of telomere length 1) has also been implicated in G4 processing [26,45]. RTEL1 is an essential helicase involved in DNA replication and recombination, and is required for the maintenance of telomere integrity (reviewed in Vannier et al., 2014 [46]). In vitro, RTEL1 is capable of unwinding G4 with a 5′–3′ polarity [47]. Remarkably, RTEL1 exhibits a PIP box domain that mediates the interaction with PCNA, suggesting that RTEL1 is recruited at telomeres by the replisome, to remove G4 [47]. Additionally, telomere fragility in a RTEL1-deficient mouse cell line is exacerbated by the loss of BLM, indicating that BLM and RTEL1 function in distinct pathways [45]. These results suggest that both the replisome through PCNA, and shelterin through TRF1, recruit specific helicases to suppress G4 structures, allowing the proper replication of telomeres.

The Pif1 helicase family is a group of 5′–3′-directed helicases found in nearly all eukaryotes. The *Saccharomyces cerevisiae* Pif1 (ScPif1) is the best-characterized member of this family. Among the multiple nuclear functions that ScPif1 fulfills in a cell (reviewed in Bochman et al., 2010 [48]), this helicase is a potent G4 unwinder and promotes replication through G4 motifs in the genome [49,50]. At telomeres, ScPif1 could probably unwind G4 [50], but presumably the main function of ScPif1 is to inhibit telomerase action by releasing the telomerase RNA from telomeric ends [51,52]. However, although the role of ScPif1 in G4 unwinding is clear, there is no evidence that ScPif1 is associated with the replisome, or binds to telomeric proteins to process telomeric G4 at telomeres. Instead, ScPif1 may preferentially patrol DNA and anchor itself to a 3′-tailed DNA junction, in order to unfold G4 [53]. Budding yeast is unique because it encodes yet another helicase, Rrm3, that belongs to the Pif1 family [48]. Unlike Pif1, Rrm3 appears to travel with the replication fork and contributes to the efficient replication of telomeric repeats [54]. Although Rrm3 is able to suppress G4-induced genome instability when the level of Pif1 is low [55], it is not established whether it is required to remove G4 at telomeres.

The human PIF1 (hPIF1) is also able to unwind G4 structures in vitro [56], and localizes to sites thought to be G4 structures [57]. Nevertheless, a direct role of hPIF1 in G4 processing at mammalian telomeres has not yet been established, although hPIF1 and mouse PIF1 seem to associate with TERT in vivo [58,59]. The unique member of the Pif1 family in the fission yeast is the helicase Pfh1 [60]. Pfh1 is essential for the replication of regions that are difficult to replicate and promotes fork movement past G4 [61,62]. At telomeres, Pfh1 facilitates telomeric DNA replication, likely by unwinding the G4 structures that are formed [63,64]. In contrast to its budding yeast counterpart Pif1, but like Rrm3, one could speculate that Pfh1 may travel with the fork, in order to facilitate telomere replication.

Despite the large number of helicases involved in G4 resolution at chromosome ends, it appears that under certain conditions, telomeric G4 can be cleaved by a nuclease. This is possibly one function of DNA2, a 5′–3′-directed helicase with a 3′-exo/endonuclease activity involved in the maintenance of genome stability [65]. Indeed, mammalian DNA2 is able to cleave G4 in vitro, and DNA2 deficiency leads to telomere replication defects. DNA2 could associate with telomeres through interaction with TRF1–TRF2, as these proteins co-immunoprecipitate [65], unless its recruitment depends on RPA [66,67]. Cleavage of G4 structures causes DNA breakage and would be expected to be deleterious for telomere replication. Thus, one might imagine that G4 cleavage is not the favored option for limiting replication stress during the replication of telomeric sequences.

### 3.2. G-Quadruplex Dissolution by Single-Strand Binding Proteins

Aside from helicases, several single-strand DNA binding proteins (SSB) have been described as preventing the formation of G4 structures in vitro. These include the telomeric protein POT1 [68,69,70], and the replication factor A complex RPA [71]. POT1, rather than being an active G4 unwinder like a helicase, would act as a steric driver that binds to telomeric tails, and then destabilizes or prevents G4 formation [70] (Figure 2). For its part, RPA is not a core component of shelterin, but would rather be brought to telomeres during replication by the incoming fork. In vitro, RPA is able to bind and unfold G4 structures according to a 5′–3′ directionality [71,72]. Indeed, RPA binds preferentially to the 5′ single-stranded tail of the G4 structure. A mutation in the DNA binding domain A of human RPA1 (D228Y) alters the DNA binding activity of the RPA complex and therefore, its G4 unwinding function [73]. In fission yeast, the corresponding mutation (D223Y) provokes the shortening of telomeres [74] and this phenotype is rescued by overexpression of the Pif1 helicase members [73]. This suggests that, in vivo, the RPA complex prevents the formation of G-rich structures at lagging strand telomeres, in order to facilitate telomerase action. Thus, it is likely that RPA unwinds G4 in vivo. Another possibility, that is not mutually exclusive with the previous one, is that RPA recruits or stimulates helicases to resolve G4 structures. Indeed, the direct physical interaction between RPA and WRN suggests that both proteins may function together in vivo [75]. Along the same lines, human replication protein A (huRPA) and *Saccharomyces cerevisiae* RPA (ScRPA) stimulate the G4 cleavage activity of huDNA2 and ScDna2, respectively [66]. RPA also interacts with FANCJ (Fanconi anemia (FA) complementation group J), a 5′–3′ helicase involved in interstrand crosslink repair (ICL) [76]. Interestingly, FANCJ is also capable of unwinding G4 structures in vitro [77,78], and this activity is stimulated by RPA [77]. In vivo, FANCJ promotes DNA synthesis through G4 structures, independently of its function in the FA pathway [79]. Despite these findings, a direct role of FANCJ at telomeres has not yet been established, although FANCJ has been identified at telomeres in ALT cells [80]. Thus, RPA might play a key role in preventing or unwinding G4 structures, either by acting alone, or by recruiting a complementary factor such as a helicase.

According to these observations, cells have evolved multiple pathways to resolve G4 structures that threaten the integrity of telomeres during replication. This complex network may act sequentially or work together to prevent the formation of secondary structures. This network would likely rely on the central component of the replisome PCNA, that may act as a tool belt for the recruitment of different replication factors, as well as the shelterin proteins TRF1 and TRF2 that may be used as a scaffold, but also POT1, the RPA complex, and probably other factors that remain to be discovered.

### 3.3. T-Loop Dissolution

The T-loop, where the 3′ overhang of telomeres invades the double-stranded part of the telomeric repeats through strand displacement to form a D-loop, participates in telomere protection, but also represents an obstacle to the progression of the replication fork. Mechanisms that dismantle the T-loop to allow the telomerase access to the 3′ overhang and to avoid collision with the replisome during the S phase are therefore necessary (Figure 2). In addition to its ability to unwind G4, RTEL1 is involved in T-loop disassembly during DNA replication [45,81]. For this function, RTEL1 recruitment requires a direct interaction with TRF2 through a C4C4 motif [82]. While it is established that RTEL1 associates with the replisome through PCNA binding to promote telomere and genome wide replication [47], the TRF2–RTEL1 interaction is likely to be tightly controlled to anticipate T-loop dissolution prior to the arrival of the replication fork. In future studies, it would be interesting to further define how RTEL1 interactions with PCNA and TRF2 are coordinated throughout the cell cycle, in order to discriminate between different replication barriers such as G4, T-loops, or other barriers. It is currently unknown whether RTEL1 itself is subject to post-translational modifications. However, potential phosphorylation sites found in TRF2 might be important for the mediation of the RTEL1–TRF2 interaction [83].

In the absence of RTEL1, T-loops are inappropriately resolved by the SLX1–SLX4 nucleases, leading to catastrophic telomere events such as t-circle formation and rapid telomere shortening [45]. However, RTEL1 might not be the sole helicase involved in T-loop disassembly. WRN and BLM are also capable of dissociating telomeric D-loop in vitro [43,84], and these two helicases are known to interact directly with TRF2 [42,43] and POT1 [85]. T-loop resolution might also involve an additional member of the RECQ helicase family, RECQL4, which is mutated in the Rothmund–Thomson syndrome [86].

The resolution of complex structures at chromosome ends relies on several helicases, while the SLX1–SLX4 nucleases seem to be used as a last resort. Indeed, the shelterin protein TRF2 seems to play a key role in recruiting and/or stimulating WRN, BLM, and RTEL1 helicases to disassemble the T-loop [42,43,82]. It is likely that many other actors involved in this process remain to be identified. Now, we need to discover how the cell orchestrates T-loop resolution when the replication fork approaches, and the nature of the cellular signal that triggers T-loop disassembly. Notably, TRF2 also functions with the Apollo 5′-exonuclease, to protect telomeres [87,88]. It is currently unknown whether Apollo is involved in T-loop resolution, nevertheless, it prevents the formation of topological constraints by the removal of superhelical stress, caused by the nucleoprotein complex that stabilizes the base of the T-loop [29]. Mechanisms that orchestrate T-loop resolution are likely to rely on a complex network of post-translational modifications, presumably involving the shelterin proteins. This may represent an active area of investigation in future.

## 4. TERRA Transcription

**Figure 3:**
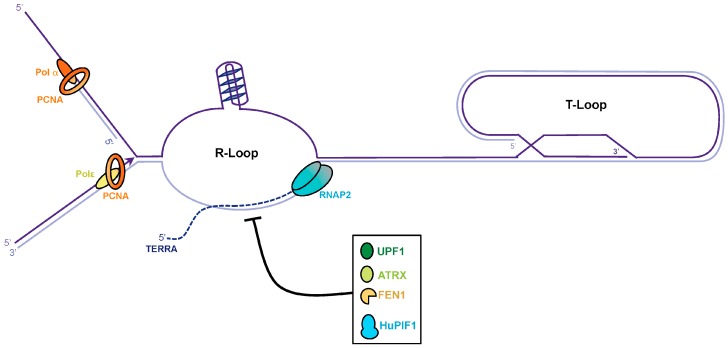
Dissolution of the telomere repeat containing RNA (TERRA) R-loop at telomeres. The C-rich telomeric strand provides the template for TERRA transcription. RNA molecules can anneal to its genomic template co-transcriptionally to generate RNA:DNAhybrids. G4 structures might form at the displaced G-rich strand and stabilize the R-loop. To avoid collisions during replication the TERRA R-loop must be dissolved. RNase H can degrade TERRA but other factors as UPF1, ATRX, FEN1, and PIF1 might also be involved in TERRA R-loop dissolution.

In most eukaryotes, telomeres are actively transcribed into TERRA [89,90]. TERRA transcription initiates from the subtelomeric regions, towards the TTAGGG tract. Under certain circumstances, RNA molecules can anneal to their genomic template co- or post-transcriptionally, in order to generate RNA:DNA hybrids. Strand displacement by the RNA:DNA hybrids forms a typical structure known as an R-loop [91]. TERRA R-loops are natural structures that are formed at telomeres in human and yeast cells, and may contribute to replication stress and chromosomal instability as they represent natural barriers to the progression of the replication fork (for review see Rippe et al., 2015 [92]). More dramatically, if G4 structures are formed at the displaced G-rich strand, the R-loop may be a major determinant of replication fork progression impairment, double-strand break, and telomeric loss. TERRA expression is cell-cycle regulated, it peaks at the G_1_–S transition and declines from S to G_2_ in telomerase-positive mammalian cells [93], and also in budding yeast [94], probably to avoid collision between RNA Pol II-mediated transcription and the replication fork. This also implies that the TERRA R-loop must be dissolved prior to the passage of the fork (Figure 3). A major enzyme involved in RNA:DNA hybrid resolution is the RNA endonuclease H (RNase H), which degrades the RNA moiety of the duplex [95]. TERRA transcription is enhanced at telomeres in the absence of telomerase, and the R-loop is mainly dissolved by RNase H in budding yeast [96], and by RNase H1 in ALT cells [97]. Moreover, *ATRX* mutations are frequently found in ALT cancer cells [98]. ATRX is a chromatin remodeler involved in the establishment of silent heterochromatin by deposition of the histone H3.3 variant in repetitive regions, such as the pericentric chromatin and telomeres [99]. Knockdown of *ATRX* results in persistent TERRA levels in G_2_/M, suggesting that ATRX promotes TERRA displacement [93]. The mechanism by which ATRX promotes TERRA displacement is currently not clear. One possibility would be that ATRX recognizes and/or modifies G4 structures by itself. Indeed ATRX has been found to bind with G4 in vitro [100]. Another hypothesis would be that ATRX influences gene expression by recognizing unusual DNA structures, converting them into regular forms by facilitating the incorporation of the histone variant H3.3 [101].

UPF1 (Up-frameshift 1) is a conserved eukaryotic phosphoprotein with nucleic acid-dependent ATPase and 5′–3′ helicase activity. UPF1 has a dual function, first in cytoplasmic RNA quality control, and second in S phase progression and genome stability [102]. At the chromosome ends, UPF1 depletion induces severe telomeric aberrations and TERRA accumulation [89]. Interestingly, the telomeric defects observed upon UPF1 depletion mainly arise from incomplete leading-strand telomere replication [103]. Taken together, these studies suggest that UPF1 participates in telomere replication, raising the possibility that UPF1 may displace TERRA molecules from telomeric chromatin.

The ability of budding yeast Pif1 and hPIF1 to unwind RNA:DNA hybrids [52,104] makes this helicase a potential candidate for resolution of the R-loop [105]. Furthermore, it has recently been suggested that the FEN1 flap endonuclease could process RNA:DNA hybrids and limit their accumulation at the leading strand [106]. These findings suggest that the replisome can bypass the RNA:DNA hybrid, to avoid co-directional collision between the replisome and RNA Pol II. The role of FEN1 would be to remove RNA:DNA hybrid flaps to avoid their accumulation, which could lead to telomere fragility.

Although TERRA transcription and the R-loop represent real threats to the replication of telomeric repeats, it becomes clear that TERRA fulfills numerous and important functions in the biology of telomeres, including telomere length regulation, telomere replication, telomere end protection, telomeric chromatin composition changes, and telomere mobility (for review [107,108,109]). Thus, telomere transcription should be tightly controlled, particularly when replication is engaged, so that collisions are avoided. In this review, we have listed several proteins that promote degradation or displacement of TERRA, but how these mechanisms are orchestrated and regulated when the replication fork approaches will require further investigation.

## 5. Telomere Compaction and Anchoring

**Figure 4:**
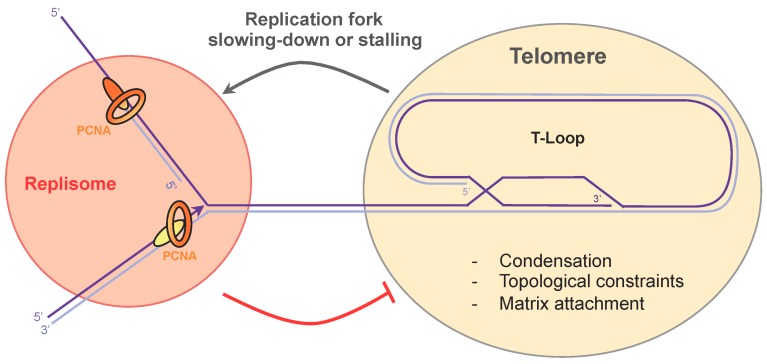
Replication fork passage through the telomeric repeat sequences. Topological constraints, condensation, and attachment to the nuclear matrix impede replication fork progression at telomeres. Cross-talk between the replisome and shelterin may take place to promote telomere decompaction and replication fork slowing-down.

Telomeres form a compact chromatin structure in vivo, through specific protein–protein and protein–DNA interactions between the shelterin proteins and telomeric DNA [110]. In line with these findings, TRF2 confers a topological state to telomeric DNA that participates in telomere protection [111]. This DNA compaction participates in the protection of the telomeric repeats by preventing DNA damage response signaling at telomeres. However, the condensation and topological constraints of the telomeric tracts also represent a barrier to the progression of the replication fork (Figure 4). This implies that, during the S phase, the telomeric DNA must be decondensed to allow passage of the replication fork, and then recompacted post-replication. This raises the question of how telomere decompaction is performed. As mentioned above, TRF2 and the 5′-exonuclease Apollo cooperate to remove superhelical constraints [29]. Moreover, they act in synergy with the DNA topoisomerase 2α, by removing the topological barrier generated by fork progression through telomeric chromatin. Thus, it seems clear that TRF2 plays an essential function in controlling the topological state of telomeric DNA. Additional work will be necessary to elucidate mechanisms that are used by the cell to regulate telomere condensation as the incoming fork approaches.

Telomere anchoring highlights another type of superhelical constraint that is introduced by tethering. In yeasts, telomeres adopt the “Rabl” conformation. Telomeres localize to the nuclear envelope (NE) on one side of the nucleus, while centromeres occupy the other side [112,113]. In budding yeast, telomere anchoring depends on the redundant Esc1–Sir4–Rap1 and yKu–Mps3 pathways. In fission yeast, NE tethering is mediated by the interaction between the inner nuclear membrane components Bqt4 and Rap1 [114]. In addition, the Fun30 chromatin remodeler Fft3 also participates in telomere anchoring, independently of Bqt4 [115]. In contrast, human telomeres localize throughout the nuclear volume. However, human telomeres are not free to roam across the nucleus, but are attached to the nuclear matrix (NM) through shelterin and lamins [116,117], and only a subset of telomeres are found at the nuclear periphery [15]. Thus, to allow efficient replication of telomeres, these topological constraints need to be released by promoting telomere detachment from NE or NM. This may represent another active area of research for the next decade.

## 6. Concluding Remarks

The passage of the replication fork through the telomeric tract is probably one of the riskiest processes that occurs during chromosome duplication. In this paper, we have reviewed the difficulties that a replication fork encounters when approaching the telomeric chromatin, and we have listed the known mechanisms that shelterin and the conventional replication machinery implement to ensure the efficient replication of telomeres. For some time now, secondary structures, such as G4 and T-loops, have been identified as natural barriers to replication at telomeres, but more recent characteristics of telomeres, including their transcription and topological constraints formed by compaction and anchoring, also have to be considered as additional sources of replication stress. Shelterin, in particular TRF1 and TRF2, plays a major role in preventing replication stress at telomeres. To date, it appears that both work in two distinct pathways; TRF1 ensures efficient replication of telomeric DNA by preventing fork stalling and activation of the S phase ATR-dependent signaling [26], while TRF2 regulates the topological constraints generated by the progression of a replication fork through telomeric chromatin [29]. How the actions of TRF1 and TRF2 are coordinated to promote efficient telomere replication will require extensive work, in order to obtain a better view of the mechanisms involved. The numerous post-translational modifications of TRF1 and TRF2 [83] might be a key feature in the regulation of these events throughout the cell cycle. Additionally, identification of molecular interactions between the replisome and the shelterin represents a challenge for the future. Integration of cellular signals to control telomere replication is likely to rely on a complex network of protein–protein interactions through post-translational protein modifications and cell cycle-dependent regulation (Figure 4).

The telomere-counting model has been proposed to explain stochastic telomere length elongation by telomerase at short telomeres [118]. This model is based on the additive negative effect of telomere-bound proteins on telomerase access to telomere. An alternative to this model, the replication fork model, has been recently proposed [119]. This alternative model takes into account the role played by replication in telomere length homeostasis. In this model, the telomerase travels with the fork and is left at the end of chromosomes to extend telomeres. Therefore, the probability that a telomere is elongated is inversely proportional to its length: short telomeres have a greater chance of being lengthened than longer ones, and vice versa. This model is compatible with the fact that the natural barriers that impede replication fork progression at telomeres have a negative impact on telomere length, presumably by providing an opportunity for the telomerase to dissociate. Consistent with this model, it has been proposed in fission yeast that a tight coordination of the leading and lagging strand DNA polymerases by shelterin limit Rad3^ATR^ accumulation and Ccq1 phosphorylation, in order to control telomerase recruitment at the chromosome ends [19]. All together, these observations may explain why mutations affecting replisome integrity or fork progression, cause telomere loss. Whatever model is closest to the real picture, it has been long realized that telomerase-dependent elongation of telomeres is intimately linked to their replication [120], and that the efficient replication of telomeres is a prerequisite for telomere elongation by telomerase.
